# Mathematical Modeling of Perfect Decoupled Control System and Its Application: A Reverse Osmosis Desalination Industrial-Scale Unit

**DOI:** 10.1155/JAMMC.2005.50

**Published:** 2005

**Authors:** C. Riverol, V. Pilipovik

**Affiliations:** 1 Chemical Engineering Department University of West Indies St. Augustine Trinidad and Tobago; 2 Project Engineering Department J. C. Engineering Los Palos Grandes Caracas Venezuela

## Abstract

This short paper outlines the computer simulation using real data of a decoupled control system for a desalination unit. The control strategy incorporated a perfect decoupled controller for the control of the fresh water flow and conductivity. The model was estimated using real data and empirical tools instead of mass balances. The success is demonstrated in the reduction of wide fluctuations in the variables of the process and decreasing of the sensibility to the changes of pressure and/or pH and allows predicting problems of quality of water and waste of energy in the future.


Journal of Automated Methods & Management in Chemistry, **2005** (2005), no. 2, 50–54
© 2005 Hindawi Publishing Corporation

### Mathematical Modeling of Perfect Decoupled Control System and Its Application: A Reverse Osmosis Desalination Industrial-Scale Unit

C. Riverol[1] and V. Pilipovik[2]

[1] *Chemical Engineering Department, University of West Indies, St. Augustine, Trinidad and Tobago*
[2] *Project Engineering Department, J. C. Engineering, Los Palos Grandes, Caracas, Venezuela*

*Received 5 February 2004; Accepted 20 February 2004*

This short paper outlines the computer simulation using real data of a decoupled control system for a desalination unit. The control strategy incorporated a perfect decoupled controller for the control of the fresh water flow and conductivity. The model was estimated using real data and empirical tools instead of mass balances. The success is demonstrated in the reduction of wide fluctuations in the variables of the process and decreasing of the sensibility to the changes of pressure and/or pH and allows predicting problems of quality of water and waste of energy in the future.

### 1. INTRODUCTION

When we try to separate pure water and a salt solution through a semipermeable membrane, the pure water diffuses through the membrane and dilutes the salt solution. The membrane rejects most of the dissolved salts, while allowing the water to permeate.

This phenomenon is known as *natural osmosis.* As water passes through the membrane, the pressure on the dilute side drops, and the pressure of the concentrated solution rises. The osmotic flux continues until an equilibrium is reached, where the net water flux through the membrane becomes zero at equilibrium; the liquid level in the saline water will be higher than that on the waterside. The amount of water passing in either direction will be equal. The hydrostatic pressure difference achieved is equal to the effective driving force causing the flow, called *osmotic pressure*. This pressure is a strong function of the solute concentration and the temperature, and depends on the type of ionic species present. Applying a pressure in excess of the osmotic pressure to the saline water section slows down the osmotic flow, and forces the water to flow from the salt solution into the waterside. Therefore, the direction of flow is reversed, and that is why this separation process is called *reverse osmosis* (Figure 1).

Continuous progress in desalination technology makes it a prime, if not the only, candidate for alleviating severe water shortages across the globe [1]. Desalination costs have been continuously decreasing over the years as a result of advances in system design and operating experience, and the associated reductions in specific unit size and specific power consumption.

The most widely used desalination processes are membrane separation via reverse osmosis (RO), and three types of thermal separation: multistage flash desalination (MSF), multiple-effect evaporation, with thermal vapor compression (MEE-TVC) and without (MEE), and mechanical vapor compression (MVC). The MSF and RO processes dominate the market for both brackish water and seawater desalination, with a total share of more than 90%. All three types of thermal desalination systems are equipped with condenser tube bundles. The MEE and MVC systems are divided into evaporating effects, while MSF systems are divided into flashing stages. All of the systems employ a number of large pumping units, including pumps for seawater intake, distillate product, brine blowdown, and chemical dosing. The MSF and MEE systems have additional pumps for the cooling seawater. In addition, MSF has pumps for brine recycle. In MSF and MEE, steam extracted from low- and medium-pressure turbine lines provides the heat necessary for flashing or evaporation. In MSF, the heating steam is routed to the brine heater; in MEE, the heating steam is routed to the first evaporating effect. The MSF process operates with a top brine temperature in the range of 90–110°C. The MEE and MVC processes are operated with lower top brine temperatures in the range of 64–70°C. MVC is distinguished from the other processes by the presence of a mechanical vapor compressor, which compresses the vapor formed within the

Correspondence and reprint requests to C. Riverol Chemical Engineering Department, University of West Indies, St. Augustine, Trinidad and Tobago; E-mail: c.riverol@eng.uwi.tt.

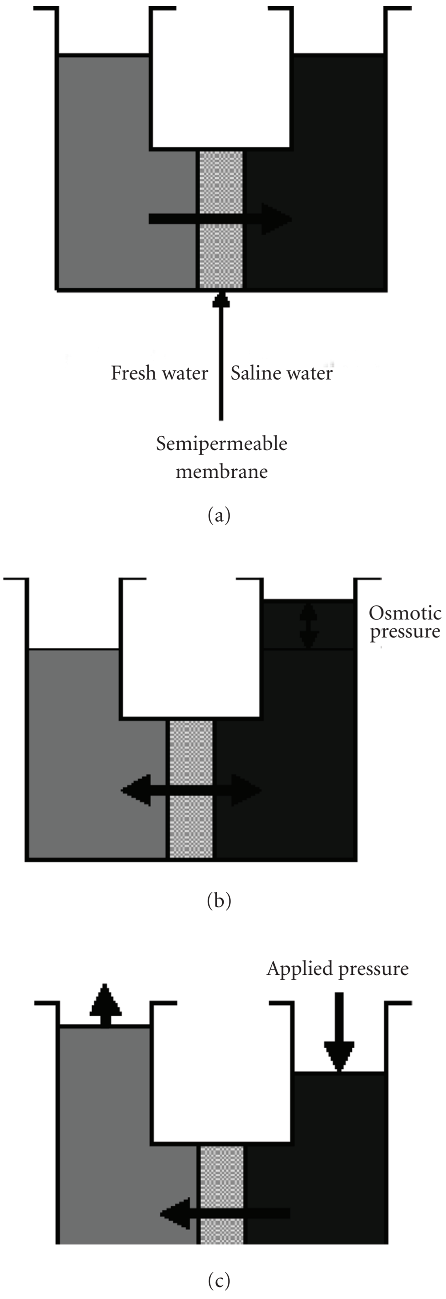

Figure 1: Principles of reverse osmosis: (a) natural osmosis, (b) osmotic equilibrium, (c) reverse osmosis.

evaporator to the desired pressure and temperature. The system also includes plate heat exchangers for preheating the feed using heat recovered from the brine blowdown stream and the distillate product.

All thermal processes produce a high-purity distillate product, with a salinity of less than 10 ppm. This is achieved by a wire-mesh mist eliminator, which removes entrained brine droplets formed in the distillate stream. The average conventional sizes are 3 000 $m^3$/d for MVC, 33 000 $m^3$/d for MSF, and 12 000 $m^3$/d for MEE. The RO process, see Figures 1 and 2, which employs membranes, has a simple layout, and is compact and modular. Existing units can be expanded to handle larger capacities. However, RO membranes are more sensitive to the conditions of the feed seawater, scaling, fouling, and pH than thermal processes. Furthermore, unlike thermal processes, RO membranes do not provide high-purity water. On average, the permeate salinity varies over a range of 30–150 ppm. The actual value depends on the process recovery, which is defined as the amount of product per unit mass of feed water. Depending on the intended use of the water, a second RO pass may be needed to reduce the salinity to an acceptable level.

The action control described in this paper is based on decoupled controllers applied to parallel transfer function processes. This method shows sensitivity to disturbance and tuning of inner loops, however the conditions were studied and the sensibility was reduced.

### 2. DESIGN OF THE CONTROL SYSTEM BASED ON EMPIRICAL MODEL

Four system parameters can be controlled in an RO unit: feed temperature, pH, conductivity, and pressure. The present study discusses two parameters which should be monitored and controlled for proper RO: conductivity (C) and inlet fresh water flow (F), and does not include the effect of the temperature over the behavior of the system. At industrial scale, the pressure affects the behavior of the system more significantly than the temperture. Additional parameters were not considered, however several literature can be consulted [2, 3] with respect other models. The manipulated variables are pH and feed pressure (P). The model was got empirically using parameter estimation. The zero/poles discrete model was obtained using real data and least square analysis and later it was transformed in continuous transfer function (s-transform). The result is similar to that in [2] although the value of the parameters is different. The system is

$$\begin{bmatrix} \mathrm{F} \\ \mathrm{C} \end{bmatrix} = \begin{bmatrix} G_{11} & G_{12} \\ G_{21} & G_{22} \end{bmatrix} \times \begin{bmatrix} \mathrm{P} \\ \mathrm{pH} \end{bmatrix}, \tag{1}$$

where

$$\begin{aligned} G_{11} &= \frac{0.0045(0.104s+1)}{0.012s^2+s+1}, & G_{12} &= \text{zero}, \\ G_{21} &= \frac{(-0.12s+0.22)}{0.1s^2+0.3s+1}, & G_{22} &= \frac{10(-3s+1)}{s^2+5s+1}. \end{aligned} \tag{2}$$

The system has to work well in a range of P = 800–1000 kPa, F = 33000–54000 $m^3$/d and pH = 6–7.2.

As in the desalination plant, a manipulated input affects more than one controlled output. One approach to handling this problem is known as decoupling [4]. The idea is to develop "synthetic" manipulated inputs that affect only one process output each. This approach is illustrated in Figure 3 where a multivariable process is controlled with a perfect decoupler and single-loop controllers with $r_1$ and $r_2$ the set points, respectively.

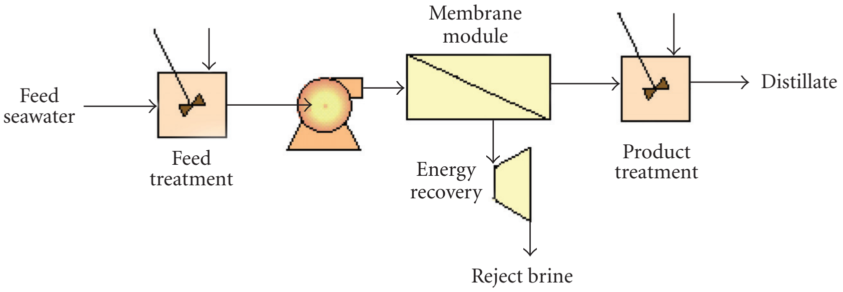

FIGURE 2: Desalination plant.

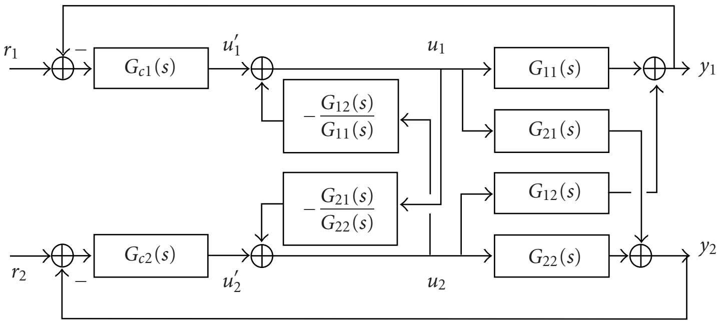

FIGURE 3: Control system.

The first step is to define the process transfer function matrix:

$$\begin{bmatrix} y_1(s) \\ y_2(s) \end{bmatrix} = \begin{bmatrix} G_{11}(s) & G_{12}(s) \\ G_{21}(s) & G_{22}(s) \end{bmatrix} \times \begin{bmatrix} u_1(s) \\ u_2(s) \end{bmatrix}$$

$$\Longrightarrow \begin{bmatrix} F \\ C \end{bmatrix} = \begin{bmatrix} G_{11} & G_{12} \\ G_{21} & G_{22} \end{bmatrix} \times \begin{bmatrix} P \\ \text{pH} \end{bmatrix}. \quad (3)$$

The perfect decoupler is built in the inverse form where each branch of the decoupler is fed before the other branch pickup point. Its transfer function is

$$\begin{bmatrix} u_1(s) \\ u_2(s) \end{bmatrix} = \frac{\begin{bmatrix} G_{11}(s)G_{22}(s) & -G_{12}(s)G_{22}(s) \\ -G_{11}(s)G_{21}(s) & G_{11}(s)G_{22}(s) \end{bmatrix}}{G_{11}(s)G_{22}(s) - G_{12}(s)G_{21}(s)} \cdot \begin{bmatrix} u_1'(s) \\ u_2'(s) \end{bmatrix}. \quad (4)$$

This decoupler is realizable only if the decoupler transfer function matrix is open-loop stable. It can be shown that the transfer function matrix of the decoupler in series with the process is

$$\begin{bmatrix} y_1(s) \\ y_2(s) \end{bmatrix} = \begin{bmatrix} G_{11}(s) & 0 \\ 0 & G_{22}(s) \end{bmatrix} \times \begin{bmatrix} u_1'(s) \\ u_2'(s) \end{bmatrix}. \quad (5)$$

For the perfect decoupler in the inverse form, single-loop controllers are tuned on the single direct transfer functions $G_{11}(s)$ and $G_{22}(s)$. Controllers are then properly tuned even if one control loop is open.

If the set points $r_1$ and $r_2$ are constrained variables, a controller structure can be used to modify the control strategy in order to cancel the $y_1$ and $y_2$ feedback and to cancel the decoupler when the constraints are not active. Inversion of the controllers at the inputs permits to reconstruct the manipulated variables $u_1$ and $u_2$ which can then be used by two other controllers to achieve control objectives with less priority than the constraints while respecting the allowable $r_1$ and $r_2$ set point values.

Pole-zero cancellation method led to the following controllers for the parallel control method:

$$G_{c1} = \frac{(0.012s^2 + s + 1)}{(0.1s + 1)}, \qquad G_{c2} = \frac{10(s^2 + 4.3s + 1)}{(-2.7s + 1)}. \quad (6)$$

The primary objective of the control system was to keep the pressure at 800 psi and the conductivity at 450 $\mu$S/cm in the face of disturbances by the pressure or/and pH. The decoupled controller has the advantage that it does not need tuning as the classical PID; only if the model is changed the controller has to be modified. With respect to the behavior of the conductivity using real data, its value is practically constant along the time using different disturbances in the pressure. A resume of the strategy is shown in Figure 4 with conductivity records collection at 5-minute intervals (22000 values) to a 30% step.

The PID control originally gave very good results with system response. This needed to be improved to remain competitive in a modern market, and has been achieved by the use of digital control. The decoupled control system offers an easy solution to the new demand of the market and it is fast implementation. It is important to note that the process has a delay time of 2.88 minutes as can be observed in

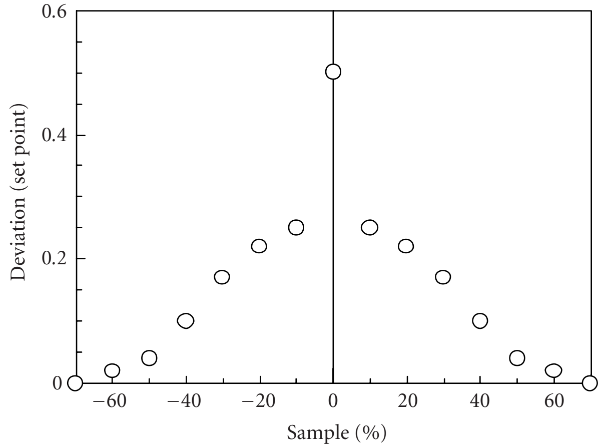

FIGURE 4: Behavior of the conductivity.

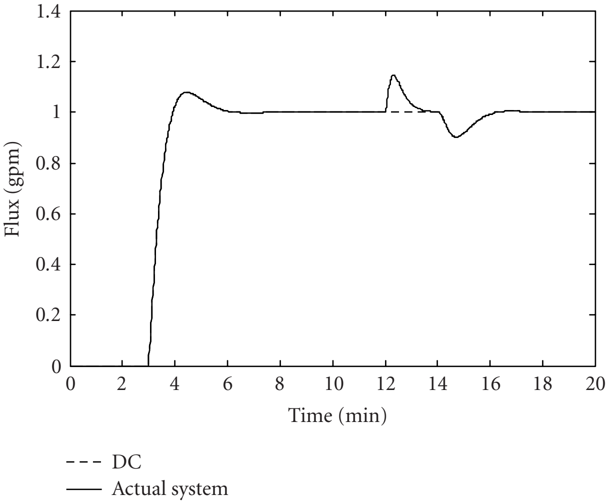

FIGURE 5: Comparison of the behavior of the water flow using both systems.

Figure 5, compared to Figure 6 that shows the behavior of one of the manipulated variables (pressure). The response is similar; however, in the classical system the response tried to become unstable or took a long time period to settle. The classical system is very sensible to any change in the manipulated variables; the use of the decoupled variables can reduce the sensibility because it tries to become the MIMO system in several SISO systems [5].

As an extension of our reasoning, if the set points $r_1$ and $r_2$ are constrained variables, a cascade controller structure can be used to modify the control strategy in order to cancel the $y_1$ and $y_2$ feedback and to cancel the decoupler when the constraints are not active. The decoupling can be achieved at the controller input with a suitable modification to the decoupler transfer functions. Inversion of the controllers at the inputs permits to reconstruct the manipulated variables $u_1$ and $u_2$ which can then be used by two other controllers to achieve control objectives with less priority than

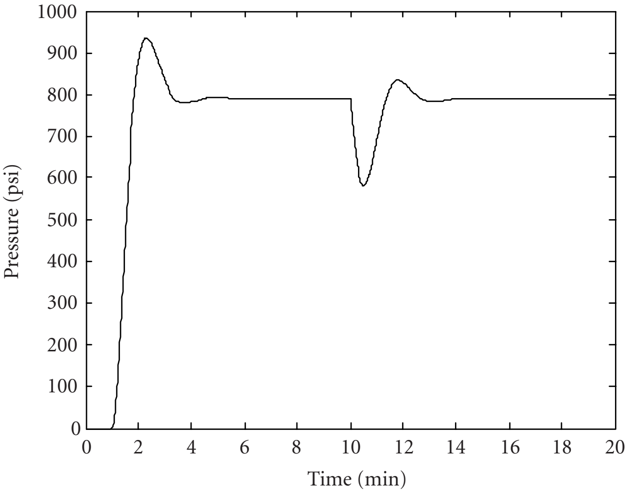

FIGURE 6: Behavior of the manipulated variable in real conditions.

TABLE 1: Resume of the results using a long-term control (ISE stands for integral square error).

| Step over acid inlet flow (%) | 10 | 20 | 30 | 40 |
|---|---|---|---|---|
| Flow rate (ISE) | 0.001 | 0.002 | 0.002 | 0.002 |
| Conductivity (ISE) | 0.001 | 0.003 | 0.007 | 0.007 |
| Trans-memb pressure (ISE) | 0.002 | 0.002 | 0.002 | 0.002 |

the constraints while respecting the allowable $r_1$ and $r_2$ set point values.

When saturation in not active, the variables $\hat{u}_i$ differ from $u_i$ only by additive constants equal to the value of the controller integrators. Therefore, the variable $\hat{u}_i$ can be used as manipulated variables by another controller to regulate additional variables.

Finally, a brief demonstration of that the decoupled control system was a success is depicted in Table 1 where the behavior of the controlled variables was evaluated using different disturbances in the pH (acid flow) in the treatment area. It is easy to observe that as the conductivity is affected directly with the changes in the pH, however the water flow is not affected for the pH, such that the conductivity may be controlled with the pH and the fresh water flow with the pressure.

### 3. CONCLUSION

A decoupled control system was developed for an RO desalination unit. Testing showed that the control system could reasonably be used for evaluating the performance of this unit. The performance of the control system was demonstrated through evaluation of some short- and long-term control strategies. Some of the interesting results obtained from this evaluation are the following: (i) there is a delay time around 2 minutes in the unit that must be considered in the design of any model or control system; (ii) in future developments for the evaluation of the long-term control strategies, benchmarks need to be able to assess settlers' performance.

### REFERENCES

[1] H. H. Ettouney, H. T. El-Dessouky, R. S. Faibish, and P. J. Gowin, "Evaluating the economics of desalination," *Chemical Engineering Progress*, vol. 98, no. 12, pp. 32–40, December 2002.

[2] I. Alatiqi, A. ghabris, and S. Ebrahim, "Measurement and control in reverse osmosis desalination," *Desalination*, vol. 75, pp. 119–140, 1989.

[3] A. Mindler and A. Epstein, "System identification and control of reverse osmosis desalination," *Desalination*, vol. 59, pp. 343–379, 1986.

[4] W. Ray, "Some recent applications of distributed parameter system theory—A survey," *Automatica*, vol. 14, pp. 281–299, 1978.

[5] J. A. Mandler, "Modeling for control analysis and design in complex industrial separation and liquefaction processes," *Journal of Process Control*, vol. 10, no. 2, pp. 167–175, 2000.

